# Combined approaches of EPR and NMR illustrate only one transmembrane helix in the human IFITM3

**DOI:** 10.1038/srep24029

**Published:** 2016-04-05

**Authors:** Shenglong Ling, Chengwei Zhang, Wei Wang, Xiaoying Cai, Lu Yu, Fangming Wu, Longhua Zhang, Changlin Tian

**Affiliations:** 1 Hefei National Laboratory of Microscale Physical Sciences, School of Life Science, University of Science and Technology of China, Hefei, 230027, P. R. China; 2 High Magnetic Field Laboratory, Chinese Academy of Sciences, Hefei, 230031, P. R. China

## Abstract

Interferon-inducible transmembrane protein IFITM3 was known to restrict the entry of a wide spectrum of viruses to the cytosol of the host. The mechanism used by the protein to restrict viral entry is unclear given the unavailability of the membrane topology and structures of the IFITM family proteins. Systematic site-directed spin labeling (SDSL) and electron paramagnetic resonance (EPR) studies of IFITM3 in detergent micelles identified a single, long transmembrane helix in the C-terminus and an intramembrane segment in the N-terminal hydrophobic region. Solution NMR studies of the same sample verified the secondary structure distribution and demonstrated two rigid regions interacting with the micellar surface. The resulting membrane topology of IFITM3 supports the mechanism of an enhanced restricted membrane hemi-fusion.

A small membrane protein family called the interferon-inducible transmembrane (IFITM) was recently discovered and is under active exploration. This family restricts a wide range of pathogenic viral infections, with different inhibitory extents for different viruses^1^,^2^,^3^. For example, IFITMs inhibit the cellular entry and replication of human immunodeficiency virus (HIV), the influenza A virus, vesicular stomatitis virus, the rabies, the West Nile virus, the dengue virus, the SARS corona virus, the Marburg virus, the Ebola virus, the Semlikiforest virus and other viruses^3^,^4^,^5^,^6^,^7^,^8^,^9^. Five members of the IFITM family have been identified in human cells, including IFITM1, IFITM2, IFITM3, IFITM5 and IFITM10^10^. Among them, IFITM1, 2 and3 can be induced by both type-1 and type-2 interferons^2^. IFITM5 cannot be induced by interferons, but it is involved in bone mineralization^11^. The detailed function of IFITM10 remains unclear^12^. IFITM2 and 3 are typically concentrated in the endosomal membrane, the lysosomal membrane or other intracellular compartments. Their subcellular distributions depend on the cell or tissue type and their expression level, but IFITM1 is expressed mainly on the plasma membrane^13^,^14^.

It is generally believed that IFITM proteins restrict viral infection by inhibiting viral membrane fusion at an early stage^6^,^15^,^16^. Recent reports have hypothesized an antiviral mechanism for IFITM proteins, suggesting that they could restrict viral membrane hemi-fusion through altering the physical properties of host cell membranes, such as reducing membrane fluidity, accumulating of cholesterol, and increasing positive spontaneous curvature in the membrane outer leaflet^16^. Moreover, post-translational modifications of IFITM3 were reported to regulate viral membrane fusion inhibition. S-palmitoylation of IFITM3 enhanced its membrane affinity and antiviral activity, whereas ubiquitination of IFITM3 decreased endo-lysosome localization and antiviral activity^17^,^18^.

Although the anti-viral functions of IFITM proteins are being comprehensively studied using variety of methods, the three-dimensional structures of IFITM proteins are not currently available. Three different membrane topology models of IFITM proteins have been proposed: an early model of dual-pass transmembrane helices with extracellular N- and C- termini (Fig. 1a, model III)^3^,^19^,^20^,^21^, a intramembrane topology model with both N-terminal domain and C-terminal domain exposing to cytoplasm (Fig. 1a, model II)^8^,^18^, and a new model with an intramembrane helix and a C-terminal transmembrane helix (Fig. 1a, model I)^22^,^23^. Therefore, further biophysical studies are urgently required to illustrate the three-dimensional structures, or at least the membrane topologies of IFITMs.

In this report, a combination of electron paramagnetic resonance (EPR) and nuclear magnetic resonance (NMR) was applied to investigate the structure and membrane topology of the IFITM3 protein in detergent micelles. Systematic site scanning of spin labeling, EPR dynamic and accessibility analysis identified a C-terminal transmembrane α-helix and an N-terminal IFITM3 segment (composed of two short α-helices) lying on the surface of micelles. Further triple resonance solution NMR studies verified the secondary structures of IFITM3 and also illustrated the backbone flexibility through NMR relaxation analysis. Collectively, a tentative IFITM3 model was proposed. This model adopts a topology similar to model I (Fig. 1a), which is consistent with recent antiviral mechanism studies.

## Results

### EPR analysis revealed the single transmembrane topology of IFITM3

**Figure 1:**
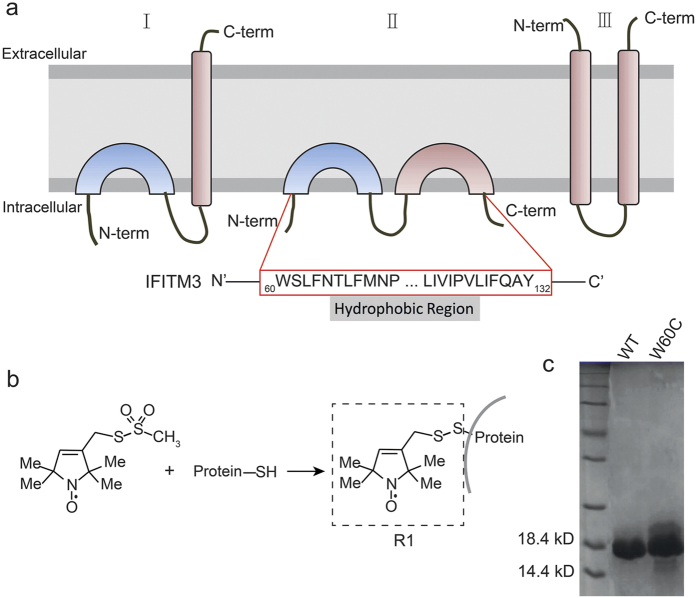
(**a**) Three different topology models proposed recently for IFITM3. The hydrophobic region of IFITM3 from W60 to Y132 was analyzed using EPR methods. (**b**) The spin labeling reaction for cysteine substituted IFITM3 mutants to introduce the nitroxide side chain, which is denoted R1. (**c**) SDS-PAGE analysis of IFITM3 in detergent micelles. Both wt-IFITM3 and IFITM3-W60C were purified as monomers in detergent micelles.

With site-directed spin labeling (SDSL), EPR spectroscopy is a powerful tool to analyze the mobility and secondary structure of a membrane protein^24^,^25^,^26^,^27^. Before implementing SDSL-EPR studies, three endogenous cysteine residues (C71, C72 and C105) on IFITM3 were first substituted with serine to construct the Cys-less “WT”. Then, a total of 72 sequential residues (from W60 to Y132, except T65, which was not expressed after cysteine mutation) covering the two predicted hydrophobic regions were mutated to cysteine residues (supplementary Figure 1). All purified IFITM3 mutants in dodecyl-phosphocholine(DPC) micelles were identified in a monomeric state using SDS-PAGE, same as the wild-type (WT) IFITM3 protein (Fig. 1c). The spin labeling reaction with methanethiosulfonate (MTSL) was subsequently performed to attach the side chain R1 to the IFITM3 mutants, showed as Fig. 1b^27^. Then, the systematic site-specific continuous wave (CW)-EPR spectra of spin-labeled IFITM3 were acquired in DPC micelles at ambient temperature(298K).

**Figure 2:**
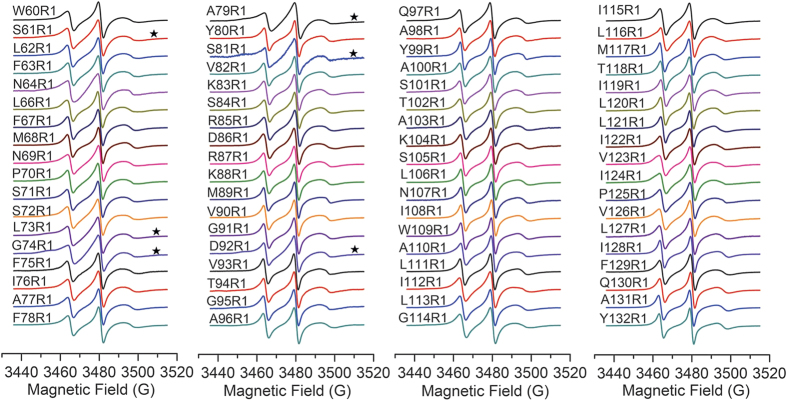
CW-EPR spectra of 72 spin-labeled Cysteine mutants, covering the hydrophobic region of human IFITM3 (form W60 to Y132, except T65). Spectra composed of multiple components were highlighted with ⁥. All spectra were normalized by the height of the central peak.

**Figure 3 f3:**
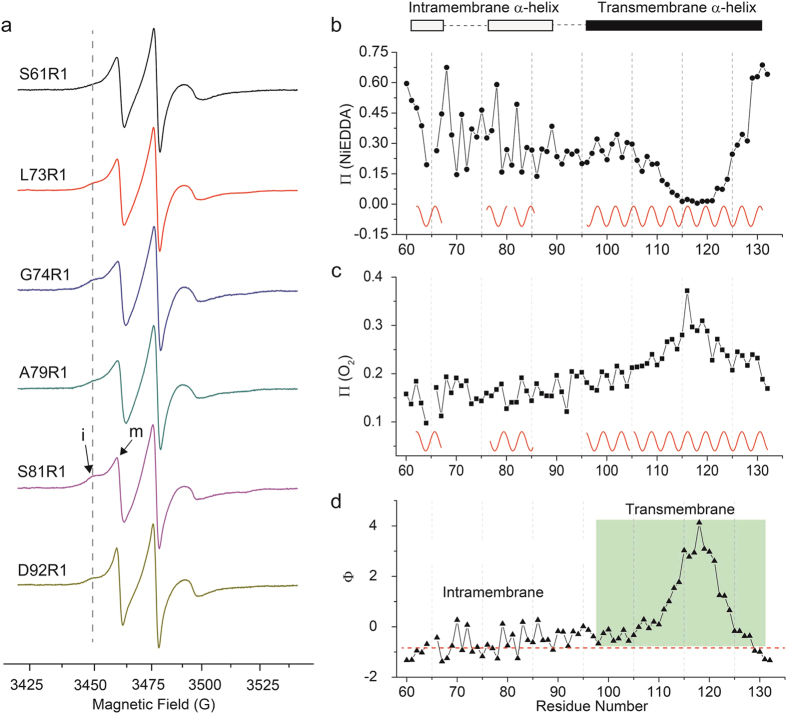
The EPR analysis of the hydrophobic region of IFITM3 in detergent micelles. (**a**) The CW-EPR spectra exhibit multi-components in detergent micelles. “i” and “m” represent the “immobile” and “mobile” components, respectively. (**b**,**c**) represent the oxygen accessibility (Π_O2_) and NiEDDA accessibility (Π_NiEDDA_) of the hydrophobic region of IFITM3 in detergent micelles. Sine waves with a periodicity of 3.6 were drawn to indicate the α-helix secondary structure. (**d**) The membrane immersion depth (Φ) of sequential residues span the hydrophobic region of IFITM3 in detergent micelles. The grey region represents the putative transmembrane region and the dashed line indicates the suggested membrane interface.

The EPR spectra line shape could reflect the mobility of the nitroxide probe R1, which also contains structural information of proteins^28^. Total of 71 CW-EPR spectra are presented in Fig. 2, and the typical nitroxide three-line CW-EPR spectra of spin-labeled IFITM3 variants were observed. Although most of the spectra exhibited only one motional state, several residue sites (S61R1, L73R1, G74R1, A79R1, S81R1 and D92R1) had multiple components. For a clear presentation, spectra of spin labels at these residue sites were plotted in Fig. 3a, indicating the presence of both the immobilized (i) and mobilized (m) components. Interestingly, all these residues with multiple motional states were identified in the first half of the putative hydrophobic region(from W60 to G95), which strongly suggests that this segment adopts two different motional or conformational states.

Then, EPR power saturation studies were conducted as shown in supplementary Figure 2. These experiments illustrate the accessibility of spin radical side chains, which can be used to derive secondary structure and the membrane topology information of proteins^24^,^29^,^30^. The systematic accessibility parameters (Π) were investigated for the sequential residues (form W60 to Y132, except T65), covering the two putative hydrophobic regions of IFITM3. Oxygen in the air was used as the hydrophobic paramagnetic reagent to evaluate membrane exposure, while NiEDDA served as the hydrophilic paramagnetic reagent to study the extent of aqueous exposure. Π_O2_ and Π_NiEDDA_ were plotted against the residue numbers in Fig. 3b,c. As we can see, the Π_NiEDDA_ values progressively decreased from A98 to G114, followed by a lower flat region (from I115 to I121). Then, the Π_NiEDDA_ values increased from residue I122 to A131. On the contrary, the value of Π_O2_ first increased starting from residue A98 and then slowly decreased. These two typical changing patterns of Π_O2_ and Π_NiEDDA_ demonstrated that a highly hydrophobic transmembrane region exists in IFITM3 starting from residue A98 to A131 in detergent micelles. However, no similar patterns in the accessibility parameters (both Π_O2_ and Π_NiEDDA_) were observed in the first half of the putative hydrophobic region (from W60 to G95), indicating that no transmembrane property exist in this segment of IFITM3. Moreover, a close examination of the accessibility parameter values (Π_O2_ and Π_NiEDDA_) of the spin labeled residues revealed some periodic behaviors. The periodicity covered the residues from A96 to A131 in Fig. 3b[Fig f3]c, indicating that this segment adopts an α-helical secondary structure in detergent micelles. In addition, the accessibility parameter values of two short segments (L62-F67 and I76-R85) in the N-terminal region were also observed with same periodicity, suggesting that two short α-helices potentially exist in this segments. Moreover, the immersion depth parameter (Φ) was derived from the conjugated accessibility parameters of Π_O2_ and Π_NiEDDA_^29^. As shown in Fig. 3d, the Φ values of the residues covering the entire putative hydrophobic region of IFITM3 can be separated into two apparently different patterns. In the first pattern starting from W60 to G95, the Φ value fluctuated around a relatively low value, indicating that the residues in this segment were partially buried in the micelles. However, the second pattern highlighted in gray (Fig. 3d) was totally different (the Φ values progressively increased from residue A98 to T118, followed by a decrease until residue Y132), indicating that a transmembrane segment was embedded in this region of IFITM3. Collectively, the analysis of the accessibility parameter (Π_O2_ and Π_NiEDDA_) and the immersion depth parameter (Φ) derived from the power saturation experiments demonstrated that the IFITM3 protein contains a long transmembrane α-helix covering the residue sequence from A96 to A131 in the putative hydrophobic region. This coincides with the previously predicted topology I and topology III model, but totally different from the topology II model (Fig. 1a). At the same time, two short α-helices without transmembrane properties were observed in the first half of the putative hydrophobic region, which was similar to the predicted topology model I or model II (Fig. 1a). Moreover, as shown in Fig. 3a, several residues in the first half of the putative hydrophobic region were revealed to contain multi-motional states by the CW-EPR spectra. Thus, our EPR analysis of IFITM3 in detergent micelles suggested that IFITM3 might adopt a membrane topology structure similar to the topology model I in Fig. 1a.

### Solution NMR structure determination and backbone relaxation analysis of IFITM3

**Figure 4:**
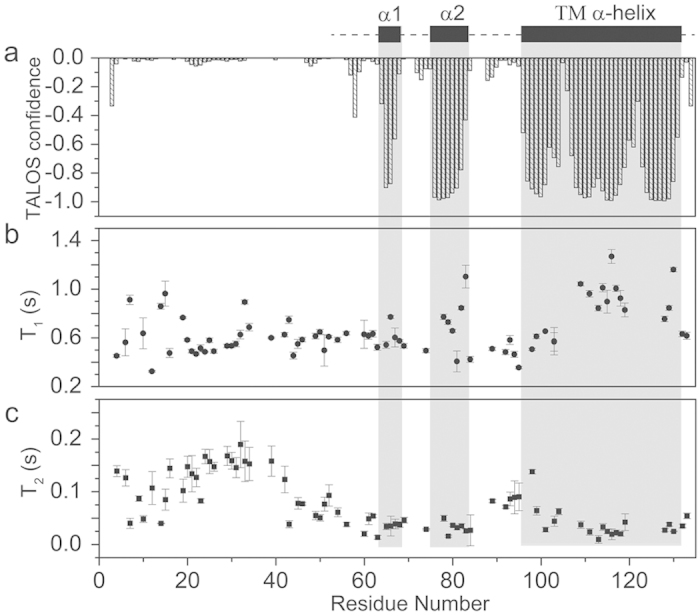
The structural analysis of IFITM3 in detergent micelles using the NMR methods. (**a**) The secondary structure of IFITM3 as predicted by the TALOS + program according to the residue assignment. (**b**,**c**) represent the longitudinal relaxation (T_1_) and transverse relaxation (T_2_) of the residue-specific backbone amide ^15^N of the IFITM3 protein in detergent micelles.

**Figure 5:**
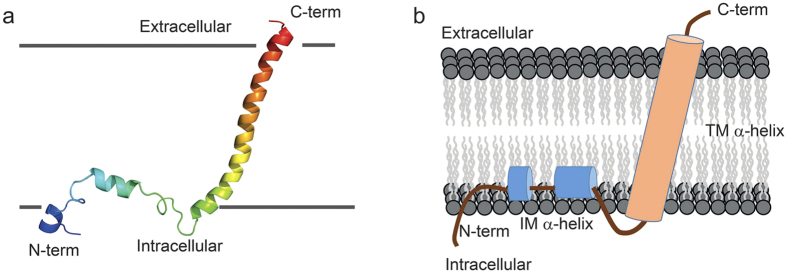
The proposed topology structure of the hydrophobic segment of IFITM3. (**a**) The structure of the hydrophobic region of IFITM3 derived from solution NMR analysis, which contains a C-terminal transmembrane α-helix and two short intramembrane α-helices. (**b**) A schematic model of IFITM3 in the membrane.

With information on the secondary structure and membrane topology of IFITM3 as determined by the EPR studies, solution NMR studies were also conducted to determine the atomic resolution structure of IFITM3 in DPC micelles. The backbone chemical shift assignments (^1^H, ^15^N, ^13^CO, ^13^C_α_, and ^13^C_β_) of full-length IFITM3 protein in DPC micelles were achieved using series of two- and three-dimensional solution NMR spectra (Fig. S3). Heteronuclear single quantum coherence (HSQC) spectra of the specifically ^15^N labeled amino acid (^15^N-Leucine, ^15^N-Isoleucine, ^15^N-Valine, ^15^N-Methionine or^15^N-Phenylalanine) in the IFITM3 samples were acquired to assist the residue assignments (Fig. S4). Based on the backbone chemical shift values of assigned residues, the program TALOS + was used to estimate the secondary structure of IFITM3^31^. As shown in Fig. 4a, three segments with relatively large negative TALOS + index values were obtained, representing three α-helix segment. The remainder of the IFITM3 protein exhibited TALOS + index values close to zero, indicating a random coil. Compared with the previously predicted topology model that contains two long hydrophobic regions, the first two short α-helixes of IFITM3 resided in the first putative hydrophobic region while the third long α-helix correspond to the second putative hydrophobic region. To further verify the secondary structure, backbone relaxation analysis of IFITM3 was performed. As shown in Fig. 4c, residues with small T2 values were mainly observed in three regions, suggesting high backbone rigidity. This finding is consistent with the α-helix structure predicted by program TALOS + in the same region. While most residues in the N-terminus have large T2 values, indicating high flexibility property of the backbone. Thus, the overall results were consistent with the derived secondary structure of IFITM3 as determined by TALOS + analysis (Fig. 4). However, the final atomic resolution structure of full-length IFITM3 could not be obtained, mainly due to the lack of an adequate number of NOE restraints and the intrinsic random coil structure in the N-terminal region. Instead, low resolution structural topology of full-length IFITM3, especially the putative hydrophobic region, can still be derived from the limited NMR data. As shown in Supplementary Figure 5, an ensembles of 10 conformers with the lowest energy and best convergence were selected from 100 calculated structures of the N-terminally truncated IFITM3 (from W60 to A131). A kink or unusual dihedral angles could be found in the C-terminal of the transmembrane helix, which were probably derived from the unassigned residues nearby and the existence of Pro125 (Figure S5). When superimposing the relatively long C-terminal α-helix (from A96 to A131), the root-mean-square deviation (RMSD) values of the backbone atoms and heavy atoms were 3.139 Å and 2.985 Å, respectively. For a clear presentation of the structure and predicted membrane topology of the putative hydrophobic region of IFITM3, one conformer was selected out (Fig. 5a). The structural model of IFITM3 shows that a long C-terminal transmembrane helix and two short intramembrane α-helices in the N-terminal of the hydrophobic region was connected by a small flexible loop between them, which is perfectly consistent with the structural topology derived from the EPR analysis. Taken together with the EPR and solution NMR results, we conclude that the hydrophobic region of IFITM3 adopts a topology containing two short intramembrane α-helices followed by a long transmembrane α-helix (Fig. 5b).

## Discussion

Three different membrane topology models for IFITM proteins have been previously proposed^14^. The first model suggested that IFITM3 adopted an intramembrane topology with both the N- and C- termini facing cytoplasm, based on the observation that the inserted N-linked glycosylation site within the N- and C-terminal region cannot be modified (Model II of Fig. 1a)^18^. The results from flow cytometry experiments and cell surface immune-staining experiments argued that IFITM3 is a dual-transmembrane protein with both its N- and C- termini exposed to the endoplasmic reticulum (ER) lumen or extracellular space (Model III of Fig. 1a)^3^,^19^. The third topology model was recently proposed demonstrating that IFITM3 is a type II transmembrane protein (model I of Fig. 1a)^22^,^23^.

Through the combined approaches of EPR and solution NMR, the structural model and the membrane topology of human IFITM3 protein were determined. As illustrated in Fig. 5b, IFITM3 adopts a transmembrane topology, supporting the proposed type II transmembrane protein topology (model I in Fig. 1a). At the C-terminus of human IFITM3, a long α-helix covering residues from A96 to A131 spans through the micelles. While two short discrete intramembrane α-helices were discovered in the first predicted hydrophobic segment of IFITM3. As shown in Fig. 3d, the EPR immersion depth (Φ) data demonstrated that these two short α-helices possessed no transmembrane properties, but only buried partially in the micelles. And the relatively larger Φ values of the residues in the second short helix indicated that it might be more deeply buried in micelles than the first short helix. Moreover, the random coil property of the N-terminus of IFITM3 was revealed by NMR analysis.

Recently, functional studies by Li *et al.* have suggested that IFITMs could hamper viral membrane hemi-fusion through reducing membrane fluidity and conferring a spontaneous curvature^16^,^32^,^33^. Here in our topology structure of IFITM3 in micelles, two short intramembrane α-helices were discovered in the first predicted hydrophobic region. These two short helices were likely to induce membrane curvature when they inserted into only one leaflet of the lipid bilayers (Fig. 5b), although lipid bilayers adopt different curvature and membrane thickness from micelles. Hence, our membrane topology of IFITM3 could provide direct structural evidence to support the antiviral mechanism proposed by Li *et al.* Moreover, three types of post-translational modifications have been reported to regulate the antiviral activity through influencing the subcellular localization, trafficking, clustering or interaction with potential receptors. The S-palmitoylation occurs at Cysteines 71, 72 and 105^17^, and ubiquitination takes place at Lysines 24, 83, 88 and 104^18^, whereas Tyrosine 20 is phosphorylated^8^,^34^. These post-translational modifications can only occur when these residues are exposed to cytoplasmic side of the membrane, which is consistent with our IFITM3 structure and proposed membrane topology model (Fig. 5b), with an intracellular N-terminal domain and an intramembrane segment facing the cytoplasm. Moreover, CW-EPR spectra analysis indicated that residues with multi-motional or multi-conformational states all resided in the intramembrane hydrophobic segments (W60-G95). We suggested that the multi-conformational property of the intramembrane hydrophobic segments might closely relate to its multiple roles in post-translational modifications, such as different structural states might interact with different enzymes.

Therefore, the combined EPR and solution NMR studies clearly supported the type II transmembrane protein topology model for IFITM3, which is consistent with the previously proposed mechanism of its antiviral function. It remains unclear why the other contradictory membrane topology models for IFITM3 are derived from functional studies. We cannot exclude the possibility that IFITM3 may be able to adopt multiple topologies in different stages of the viral infection period, or in different host cell types. Further structural and functional correlation studies of IFITM3 in different cell types and studies focusing on other IFITM proteins are still required to completely unveil the antiviral mechanism of IFITM proteins.

## Methods

### Constructions of Cysteine mutants ofIFITM3

Three endogenous cysteines (C71, C72 and C105) were substituted by Serine through site-directed mutagenesis to construct Cysless “WT” IFITM3. Then, 72 sequential residues spanning from W60 to Y132 (except T65) on full-length IFITM3 were mutated to Cysteine, respectively. The target IFITM3 gene sequences were subsequently inserted into the vector pET21b (Novagen) using the restriction sites *Nde I* and *Xho I*. All constructs were verified through gene sequencing.

### Protein expression and purification

IFITM3 protein was over-expressed in BL21-GOLD (DE3) competent cells (Novagen) transformed with vector pET21b containing IFITM3 coding sequence in M9 media at 25 °C. A final concentration of 0.8 mM IPTG (isopropyl-D-thio-galactoside) was added at cell density around OD_600_ = 0.8 to induce protein over-expression in the following 20 hours. The cell pellets harvested by high-speed centrifugation (Allegrax-15R, Beckman) were resuspended in lysis buffer(70 mM Tris, 300 mM NaCl, pH 8.0). Then, cell membrane was broken using ultrasonication, followed by separation procedure using high-speed centrifugation. After that, the pallets fraction containing IFITM3 protein was dissolved using the binding buffer (20 mM Tris, 200 mM NaCl, pH 8.0) containing 1% (w/v)SDS. Finally, IIFITM3 protein was purified using Ni-NTA affinity chromatography (QIAgen, Germany) and eluted in binding buffer containing 0.5% DPC.SDS-PAGE (Sodium dodecyl sulfate polyacrylamide gel electrophoresis) was applied to analyze the purified IFITM3 protein in DPC micelles.

### MTSL (1-oxyl-2,2,5,5-tetramethyl-Δ3-pyrr-oline-3-methyl methanethiosulfonate) spin labeling

Spin labeling reaction was carried out through incubating purified IFITM3 mutants with 10 fold molar ratio MTSL (Toronto Research Chemicals, Ontario, Canada) at 4 °C overnight. Excessive free MTSL was removed using a PD-10 gravity flow desalting column (GE Biosciences). Then, spin labeled IFITM3 protein in binding buffer with 0.2% DPC (w/v)were concentrated to approximately 200 μM using Amicon Ultra-15 centrifugal filter units (Millipore) for further EPR experiments.

### Continuous wave EPR (CW-EPR) spectroscopy

CW-EPR spectra were acquired using a Bruker A300 spectrometer (Bruker Biospin GmbH, Rheinstetten, Germany) at X-band (9.5 GHz) equipped with a high-sensitivity cavity (ER 4119HS, Bruker Biospin GmbH, Rheinstetten, Germany). Samples were placed into a quartz capillary tube(about 0.5 mm, Kimble micro-capillary pipets) with a volume of approximately 20 μL. Experimental parameters were optimized with 100 kHz modulation frequency, 2 mW incident microwave power, 1Gauss modulation amplitude, 10.24 ms time constant, 40.96 ms conversion time and 150 Gauss scan width.

For power saturation studies and accessibility analysis, the experiments were performed using the same spectrometer equipped with an ER4123D CW resonator (Bruker BioSpin, Germany). Samples with a total volume of ~3 μL were loaded into gas permeable TPX capillary tubes. During the power saturation analysis, the range of incident microwave power was from 0.7 mW (25dB attenuation) to180 mW(1dB attenuation) with the step of 2dB.To obtain the EPR spectra of IFITM3 sample in the N_2_ atmosphere, O_2_ atmosphere of the sample was purged through N_2_ blowing or equilibration. At the same time, EPR spectra were acquired in air (O_2_ as the hydrophobic paramagnetic reagent), or in N_2_ with the presence of 50 mM Ni^2+^-EDDA complex (NiEDDA) as the hydrophilic paramagnetic reagent. Power saturation curves were measured as the vertical peak-to-peak amplitude(A) of the first derivative M_i = 0_ line as a function of incident microwave power (P). To determine the value of P_1/2_, the data were fitted using an R software script according to the equation (1):


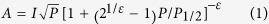


I is a scaling factor, P_1/2_ is the incident power where the first derivative amplitude is reduced to half of its unsaturated value, and ε is a measure of the homogeneity of saturation of the resonance line. With the obtained P_1/2_, the accessibility parameter Π_O2_ and Π_NiEDDA_ were calculated according to the equation (2):


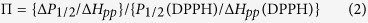


where ΔP_1/2_ is the value difference between two P_1/2_ values in the presence and absence of relaxing agent, ΔHpp is the peak-to-peak line-width of the first derivative spectrum, P_1/2_(DPPH) and ΔHpp (DPPH) were the P_1/2_ and peak-to-peak line-width values of a standard sample of crystalline 2,2-diphenyl-1-picrylhydrazyl (DPPH) in KCl.

Then, the immersion depth parameter Φ could be calculated using the power saturation parameter:


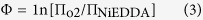


### NMR spectroscopy and structure determination

IFITM3 proteinfor NMR experiments were expressed and purified as described before, except that the final NMR samples were prepared in 50 mM phosphate buffer containing 10% D_2_O, with a concentration of 1.0 mM. A series of TROSY-based multi-dimensional NMR experiments were performed on 500 MHz Varian spectrometer, 700 MHz Varian spectrometer and 600 MHz Bruker spectrometer. ^1^H/^15^N labeled IFITM3 protein was used for 2D TROSY-HSQC and NOESY-HSQC experiments. The ^13^C/^15^N labeled sample and ^2^H/^13^C/^15^N labeled sample in DPC detergent micelles were both applied for 3D HNCA, HN(CO)CA, HNCO, HN(CA)CO, CBCANH, and CBCA(CO)NH spectra collecting at pH 7.0 and 35 °C. Besides, to assist assignment, 2D TROSY experiments were performed using ^15^N-Leucine, ^15^N-Isoleucine, ^15^N-Valine, ^15^N-Methionine and ^15^N-Phenylalanine selectively labeled IFITM3 samples. Details of the experimental parameters were described previously. All the spectra were analyzed using NMRPipe and NMRView. Backbone resonance assignment was then performed. Dihedral angle constraints were predicted using Talos + program, based on the obtained chemical shifts of ^13^Cα, ^13^Cβ, ^13^CO, ^1^Hα, ^15^N, ^1^HN. Finally, the resulting dihedral angle and NOE restrains were applied in structure calculation using Xplor-NIH program.

### Backbone ^15^N relaxation measurements of IFITM3

2D 15N-1HHSQC pulse sequences were applied in the T1 and T2 relaxation measurements. Delay values used for T1 and T2 experiments were 20, 50, 100, 200, 500, 1000 and 1500 ms, and 10, 30, 50, 90, 130 and 190 ms, respectively.

## Additional Information

**How to cite this article**: Ling, S. *et al.* Combined approaches of EPR and NMR illustrate only one transmembrane helix in the human IFITM3. *Sci. Rep.* **6**, 24029; doi: 10.1038/srep24029 (2016).

## Supplementary Material

Supplementary Information

## References

[b1] Friedman R. L., Manly S. P., McMahon M., Kerr I. M. & Stark G. R. Transcriptional and posttranscriptional regulation of interferon-induced gene expression in human cells. Cell 38, 745–55 (1984). 6548414 10.1016/0092-8674(84)90270-8

[b2] Lewin A. R., Reid L. E., McMahon M., Stark G. R. & Kerr I. M. Molecular analysis of a human interferon-inducible gene family. Eur J Biochem 199, 417–23 (1991). 1906403 10.1111/j.1432-1033.1991.tb16139.x

[b3] Brass A. L. *et al.* The IFITM proteins mediate cellular resistance to influenza A H1N1 virus, West Nile virus, and dengue virus. Cell 139, 1243–54 (2009). 20064371 10.1016/j.cell.2009.12.017 PMC2824905

[b4] Jiang D. *et al.* Identification of three interferon-inducible cellular enzymes that inhibit the replication of hepatitis C virus. Journal of Virology 82, 1665–1678 (2008). 18077728 10.1128/JVI.02113-07 PMC2258705

[b5] Weidner J. M. *et al.* Interferon-induced cell membrane proteins, IFITM3 and tetherin, inhibit vesicular stomatitis virus infection via distinct mechanisms. J Virol 84, 12646–57 (2010). 20943977 10.1128/JVI.01328-10 PMC3004348

[b6] Huang I. C. *et al.* Distinct patterns of IFITM-mediated restriction of filoviruses, SARS coronavirus, and influenza A virus. PLoS Pathog 7, e1001258 (2011). 21253575 10.1371/journal.ppat.1001258 PMC3017121

[b7] Schoggins J. W. *et al.* A diverse range of gene products are effectors of the type I interferon antiviral response. Nature 472, 481–5 (2011). 21478870 10.1038/nature09907 PMC3409588

[b8] Jia R. *et al.* The N-terminal region of IFITM3 modulates its antiviral activity by regulating IFITM3 cellular localization. J Virol 86, 13697–707 (2012). 23055554 10.1128/JVI.01828-12 PMC3503121

[b9] Chutiwitoonchai N. *et al.* Characteristics of IFITM, the newly identified IFN-inducible anti-HIV-1 family proteins. Microbes and Infection 15, 280–290 (2013). 23376165 10.1016/j.micinf.2012.12.003 PMC7110712

[b10] Siegrist F., Ebeling M. & Certa U. The small interferon-induced transmembrane genes and proteins. J Interferon Cytokine Res 31, 183–97 (2011). 21166591 10.1089/jir.2010.0112

[b11] Moffatt P. *et al.* Bril: a novel bone-specific modulator of mineralization. J Bone Miner Res 23, 1497–508 (2008). 18442316 10.1359/jbmr.080412

[b12] Hickford D. E., Frankenberg S. R., Shaw G. & Renfree M. B. Evolution of vertebrate interferon inducible transmembrane proteins. BMC Genomics 13, 155 (2012). 22537233 10.1186/1471-2164-13-155 PMC3424830

[b13] Diamond M. S. & Farzan M. The broad-spectrum antiviral functions of IFIT and IFITM proteins. Nat Rev Immunol 13, 46–57 (2013). 23237964 10.1038/nri3344 PMC3773942

[b14] Smith S., Weston S., Kellam P. & Marsh M. IFITM proteins-cellular inhibitors of viral entry. Curr Opin Virol 4, 71–7 (2014). 24480526 10.1016/j.coviro.2013.11.004 PMC7185728

[b15] Feeley E. M. *et al.* IFITM3 inhibits influenza A virus infection by preventing cytosolic entry. Plos Pathog 7, e1002337 (2011). 22046135 10.1371/journal.ppat.1002337 PMC3203188

[b16] Li K. *et al.* IFITM proteins restrict viral membrane hemifusion. Plos Pathog 9, e1003124 (2013). 23358889 10.1371/journal.ppat.1003124 PMC3554583

[b17] Yount J. S. *et al.* Palmitoylome profiling reveals S-palmitoylation-dependent antiviral activity of IFITM3. Nat Chem Biol 6, 610–4 (2010). 20601941 10.1038/nchembio.405 PMC2928251

[b18] Yount J. S., Karssemeijer R. A. & Hang H. C. S-palmitoylation and ubiquitination differentially regulate interferon-induced transmembrane protein 3 (IFITM3)-mediated resistance to influenza virus. J Biol Chem 287, 19631–41 (2012). 22511783 10.1074/jbc.M112.362095 PMC3365998

[b19] Takahashi S., Doss C., Levy S. & Levy R. Tapa-1, the Target of an Antiproliferative Antibody, Is Associated on the Cell-Surface with the Leu-13 Antigen. Journal of Immunology 145, 2207–2213 (1990). 2398277

[b20] Evans S. S., Lee D. B., Han T., Tomasi T. B. & Evans R. L. Monoclonal antibody to the interferon-inducible protein Leu-13 triggers aggregation and inhibits proliferation of leukemic B cells. Blood 76, 2583–93 (1990). 2265250

[b21] Bailey C. C., Zhong G., Huang I. C. & Farzan M. IFITM-Family Proteins: The Cell’s First Line of Antiviral Defense. Annu Rev Virol 1, 261–283 (2014). 25599080 10.1146/annurev-virology-031413-085537 PMC4295558

[b22] Weston S. *et al.* A membrane topology model for human interferon inducible transmembrane protein 1. Plos One 9, e104341 (2014). 25105503 10.1371/journal.pone.0104341 PMC4126714

[b23] Bailey C. C., Kondur H. R., Huang I. C. & Farzan M. Interferon-induced transmembrane protein 3 is a type II transmembrane protein. J Biol Chem 288, 32184–93 (2013). 24067232 10.1074/jbc.M113.514356 PMC3820858

[b24] Yu L. *et al.* CW-EPR studies revealed different motional properties and oligomeric states of the integrin beta1a transmembrane domain in detergent micelles or liposomes. Sci Rep 5, 7848 (2015). 25597475 10.1038/srep07848 PMC4297981

[b25] Claxton D. P. *et al.* Ion/substrate-dependent conformational dynamics of a bacterial homolog of neurotransmitter: sodium symporters. Nat Struct Mol Biol 17, 822–9 (2010). 20562855 10.1038/nsmb.1854 PMC3245867

[b26] Perozo E., Cortes D. M. & Cuello L. G. Three-dimensional architecture and gating mechanism of a K+ channel studied by EPR spectroscopy. Nat Struct Biol 5, 459–69 (1998). 9628484 10.1038/nsb0698-459

[b27] Hubbell W. L., McHaourab H. S., Altenbach C. & Lietzow M. A. Watching proteins move using site-directed spin labeling. Structure 4, 779–83 (1996). 8805569 10.1016/s0969-2126(96)00085-8

[b28] McHaourab H. S., Lietzow M. A., Hideg K. & Hubbell W. L. Motion of spin-labeled side chains in T4 lysozyme. Correlation with protein structure and dynamics. Biochemistry 35, 7692–704 (1996). 8672470 10.1021/bi960482k

[b29] Altenbach C., Greenhalgh D. A., Khorana H. G. & Hubbell W. L. A collision gradient method to determine the immersion depth of nitroxides in lipid bilayers: application to spin-labeled mutants of bacteriorhodopsin. Proc Natl Acad Sci USA 91, 1667–71 (1994). 8127863 10.1073/pnas.91.5.1667 PMC43224

[b30] Zhou Z. *et al.* Structure of the cytoplasmic domain of erythrocyte band 3 hereditary spherocytosis variant P327R: band 3 Tuscaloosa. Biochemistry 46, 10248–57 (2007). 17696498 10.1021/bi700948p

[b31] Shen Y., Delaglio F., Cornilescu G. & Bax A. TALOS+: a hybrid method for predicting protein backbone torsion angles from NMR chemical shifts. J Biomol NMR 44, 213–23 (2009). 19548092 10.1007/s10858-009-9333-z PMC2726990

[b32] McMahon H. T. & Gallop J. L. Membrane curvature and mechanisms of dynamic cell membrane remodelling. Nature 438, 590–6 (2005). 16319878 10.1038/nature04396

[b33] Chernomordik L. V. & Kozlov M. M. Mechanics of membrane fusion. Nature Structural & Molecular Biology 15, 675–683 (2008). 10.1038/nsmb.1455 PMC2548310 18596814

[b34] John S. P. *et al.* The CD225 domain of IFITM3 is required for both IFITM protein association and inhibition of influenza A virus and dengue virus replication. J Virol 87, 7837–52 (2013). 23658454 10.1128/JVI.00481-13 PMC3700195

