# Supplementary material for: Combined approaches of EPR and NMR illustrate only one transmembrane helix in the human IFITM3
Source: Sci Rep. 2016 Apr 5;6:24029. doi: 10.1038/srep24029 (PMC4820770; doi:10.1038/srep24029)

Combined approaches of EPR and NMR illustrate only one transmembrane helix in the human IFITM3

Shenglong Ling[1,#],Chengwei Zhang[1,#], Wei Wang[1], Xiaoying Cai[1], Lu Yu[2], Fangming Wu[2], Longhua Zhang[1,*], Changlin Tian[1,2,*]

[1] Hefei National Laboratory of Microscale Physical Sciences, School of Life Science, University of Science and Technology of China, Hefei, 230027, P. R. China.
[2] High Magnetic Field Laboratory, Chinese Academy of Sciences, Hefei, 230031, P. R. China

[#] These authors contributed equally to this work.
[*]Correspondence should be addressed to L.Z. or C.T. (zlhustc@ustc.edu.cn or cltian@ustc.edu.cn)

Supporting Figures and Tables:

Figure S1. Primary sequence and topology diagram of human IFITM3 protein. Two transmembrane helices (orange cylinder) were predicted by software TMHMM. Residues covering the hydrophobic region (from W60 to Y132) were selected for systematic EPR studies.

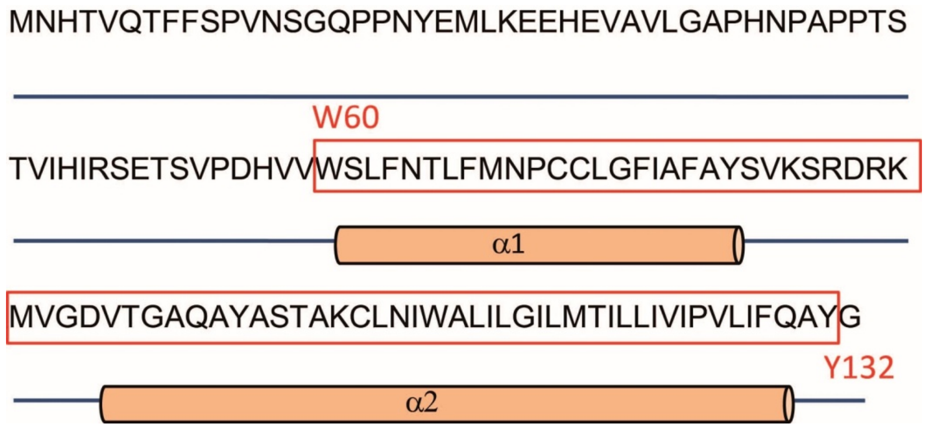

Figure S2. EPR power saturation curves for IFITM3 L113R1 in DPC micelles. The power saturation data was collected under three conditions: molecular oxygen(O_2), nitrogen (as a control) and the reagent NiEDDA (50 mM). Π_{O2} and Π_{NiEDDA} values from fitting were presented.

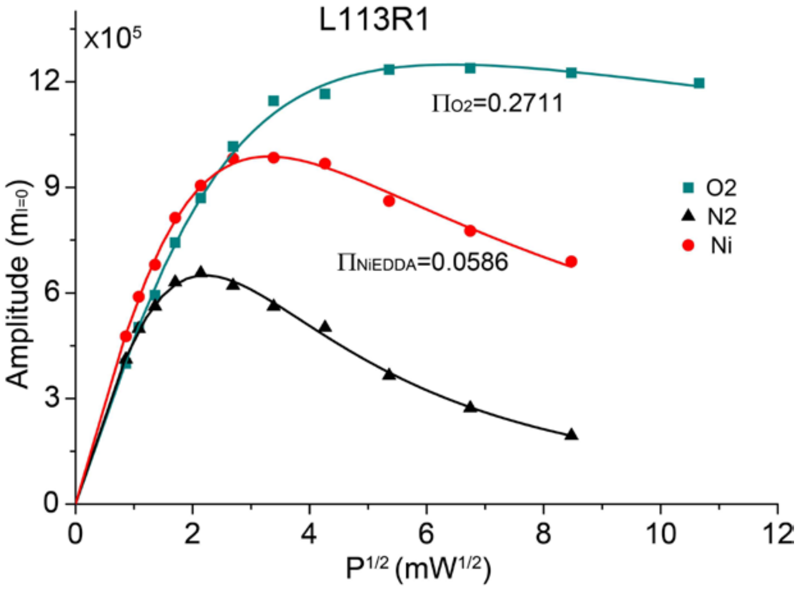

Figure S3. Backbone resonances assignmentof IFITM3 on two dimensional ^{1}H-^{15}N correlation spectra in DPC detergent micelles.

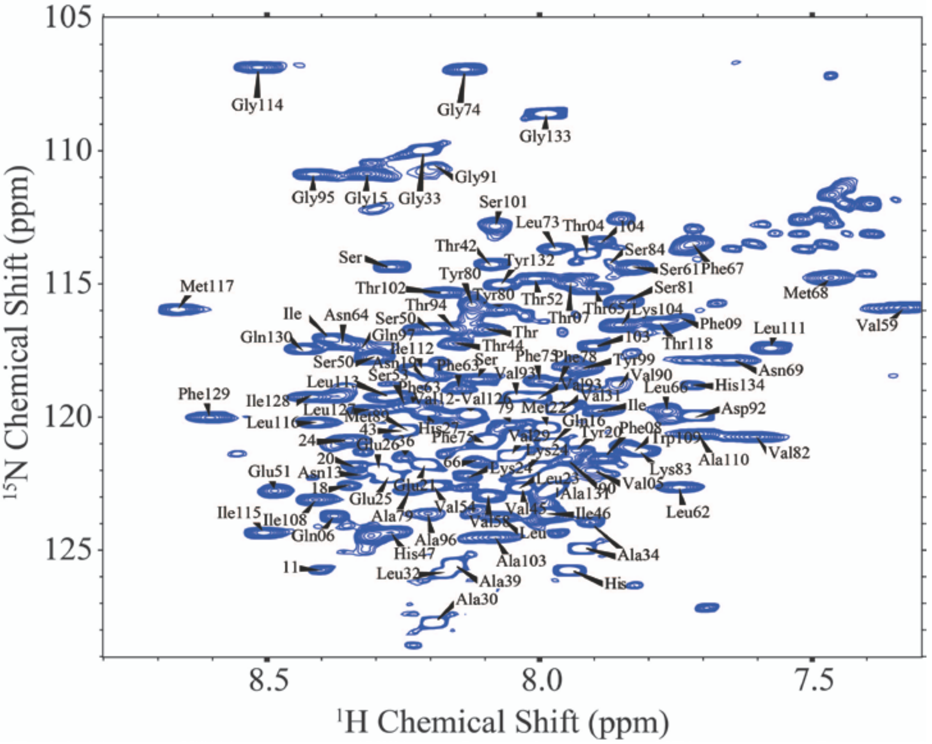

Figure S4. Two dimensional ^{15}N-^{1}H TROSY spectra of single amino acid selectively labeled IFITM3 in detergent micelles.

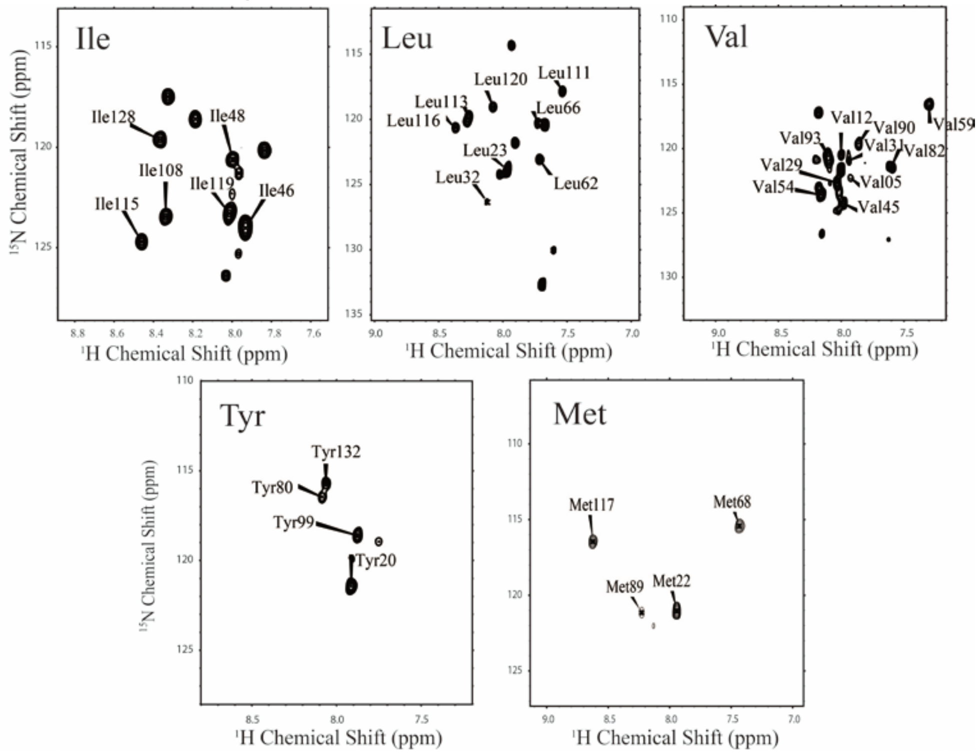

Figure S5. Structure superimposition of the final 10 conformers with the lowest energy and best convergence of the N-termini truncated IFITM3(form W60 to A131).

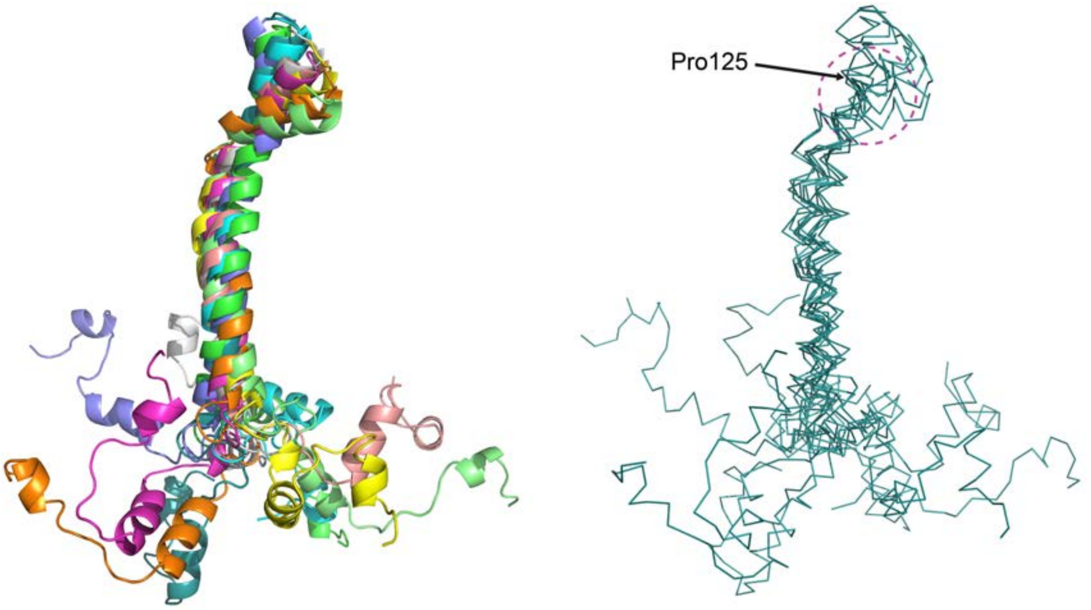

Supplement: Supplementary Information [file srep24029-s1.pdf]
